# Cancer-associated fibroblasts: heterogeneity, tumorigenicity and therapeutic targets

**DOI:** 10.1186/s43556-024-00233-8

**Published:** 2024-12-16

**Authors:** Keke Lv, Tianlin He

**Affiliations:** Department of Hepatopanreatobiliary Surgery, Changhai Hospital, 168 Changhai Road, Yangpu District, Shanghai, 200433 China

**Keywords:** Cancer associated fibroblasts, Tumor microenvironment, Extracellular matrix, Immune cells, Mechanisms, Cancer cell, Cancer targeting therapy

## Abstract

Cancer, characterized by its immune evasion, active metabolism, and heightened proliferation, comprises both stroma and cells. Although the research has always focused on parenchymal cells, the non-parenchymal components must not be overlooked. Targeting cancer parenchymal cells has proven to be a formidable challenge, yielding limited success on a broad scale. The tumor microenvironment(TME), a critical niche for cancer cell survival, presents a novel way for cancer treatment. Cancer-associated fibroblast (CAF), as a main component of TME, is a dynamically evolving, dual-functioning stromal cell. Furthermore, their biological activities span the entire spectrum of tumor development, metastasis, drug resistance, and prognosis. A thorough understanding of CAFs functions and therapeutic advances holds significant clinical implications. In this review, we underscore the heterogeneity of CAFs by elaborating on their origins, types and function. Most importantly, by elucidating the direct or indirect crosstalk between CAFs and immune cells, the extracellular matrix, and cancer cells, we emphasize the tumorigenicity of CAFs in cancer. Finally, we highlight the challenges encountered in the exploration of CAFs and list targeted therapies for CAF, which have implications for clinical treatment.

## Introduction

Solid tumors, such as pancreatic cancer, breast cancer, lung cancer, and colorectal cancer, which are characterized by high fibrosis and heterogeneity, have consistently seen a rise in prevalence in recent years. The prevalence of pancreatic cancer, colloquially known as the “King of Cancers,” continues to surge worldwide. Within this category, exocrine gland tumors constitute approximately 95% of pancreatic cancer cases, with pancreatic ductal adenocarcinoma (PDAC) being the predominant type [1]. Globally, pancreatic cancer ranks seventh among causes of cancer-related deaths. Despite significant variations in incidence rates among countries, the global trend shows an increasing diagnosis rate of PDAC [2]. In developed countries, the occurrence of pancreatic cancer is escalating annually at a rate of 0.5% to 1.0%. Forecasts indicate that by 2030, it will ascend to the second leading cause of cancer death in the United States while maintaining its position as the primary underlying cause [3]. Statistics indicate that pancreatic cancer predominantly affects individuals over 40 years old. However, recently, there is a trend of its incidence among young people [4]. Notably, the cure rates of breast cancer (BC), lung cancer (LC), and colorectal cancer (CRC) have undergone notable advancements due to the introduction of early screening programs. However, due to the complex and atypical nature of tissue cells and the significant presence of non-malignant components, single-modality treatment is unlikely to yield the desired results. Consequently, it becomes imperative to incorporate additional adjuvant therapies to surmount the inherent constraints of the treatment paradigm [5–8].

Cancer arises from the gradual accumulation of genetic mutations, leading to instability within the affected cells [9, 10]. The tumor microenvironment(TME), characterized by varying degrees of hypoxia and inflammation, undergoes intricate crosstalk between its malignant and non-malignant constituents, fostering an adaptive response to the dynamic alterations in cancer cell activity [11–13]. In recent years, there has been a growing trend for research to shift its focus from the tumor parenchyma towards the tumor mesenchyme, emphasizing the pivotal role played by non-malignant components. Specifically, a type of non-malignant cell that lacks markers for epithelial cells, endothelial cells, and white blood cells, exhibits a slender morphology, and is devoid of mutated oncogenes is classified as a cancer-associated fibroblast(CAF) [14].

Researchers have extensively discovered CAFs in solid tumors and have developed an interest in elucidating their pivotal role [15–17]. However, the abundance of CAF subtypes and phenotypic instability, coupled with the lack of specific markers, makes isolation and extraction of CAFs difficult. Therefore, this paper begins by introducing the heterogeneity of CAFs including their origin, subtypes and functions. While the role of CAFs has been extensively studied, there remains a notable gap in comprehensive research on the crosstalk between CAFs and various components. Particularly, in this paper, we focus on summarizing the mechanisms that drive diverse biological activities in cancer. Our primary aim is to clarify the tumorigenicity of CAFs by intricate network that encompasses cancer-associated fibroblasts (CAFs), immune cells, and cancer cells. Subsequently, we propose the double-edged role of CAFs in cancer development and suggest the necessity of its targeting. Lastly, we showcase medicine advancements aimed at CAFs, underscore the imperative for targeted therapeutic strategies, and reflect on the clinical treatment landscape. In the final summary, we discuss the current situation of CAFs from the cellular level to the macroscopic level of cancer. Concurrently, the potential opportunities with challenges, future research directions and clinical value of CAFs are also proposed.

## Heterogeneity of CAFs in cancer

CAFs are a heterogeneous population of cells originating from a wide range of cells, which are prevalently found in diverse solid tumors and are activated via multiple intricate signaling pathways. The diversity of CAFs origin and activation may gives rise to a range of phenotypes, which in turn leads to functional heterogeneity.

### Heterogeneity of origin and subtypes

CAFs are a heterogeneous population of cells originating from a wide range of cells, which are prevalently found in diverse solid tumors and are activated via multiple intricate signaling pathways. Phillip M. Galbo, Jr. harnessed an extensive dataset comprising over 10,000 RNA-seq samples from 31 distinct cancer types to classify pan-cancer CAFs (pan-CAFs) into six precise subtypes. This paper predominantly delves into two of these categories: myofibroblastic CAFs(myCAFs) and inflammatory CAFs (iCAFs) [18]. myCAFs and iCAFs were initially identified by Öhlund et al. The former are enriched near tumor cells, while the latter are distributed far from the tumor center [19]. While CAFs within various solid tumors can be categorized into the two classifications mentioned above, a growing body of research has uncovered distinct molecular expression discrepancies among CAFs through comparative analysis, thereby delineating molecular subtypes of CAFs [20].

In pancreatic cancer, tissue-resident fibroblasts are converted to CAFs due to the interplay of growth factors, chemokines, and metabolites secreted by cancer cells, alongside the influence of extracellular matrix inflammatory cells, senescent fibroblasts, and force transducers. This intricate process results in the reprogramming of fibroblasts playing a pivotal role in the tumor microenvironment [21]. Pancreatic stellate cells(PSCs) activated by transforming growth factor(TGF-β) and PDGF express α-smooth muscle actin(α-SMA), a surface marker for CAFs, and become a significant source of CAFs. However, some researchers have proposed that PSC produces only a tiny amount of CAFs [22]. In addition, several potential sources of CAFs include endothelial cells, epithelial cells, adipocytes, pericytes, smooth muscle cells, and bone marrow-derived mesenchymal cells [23–25].

**Fig. 1:**
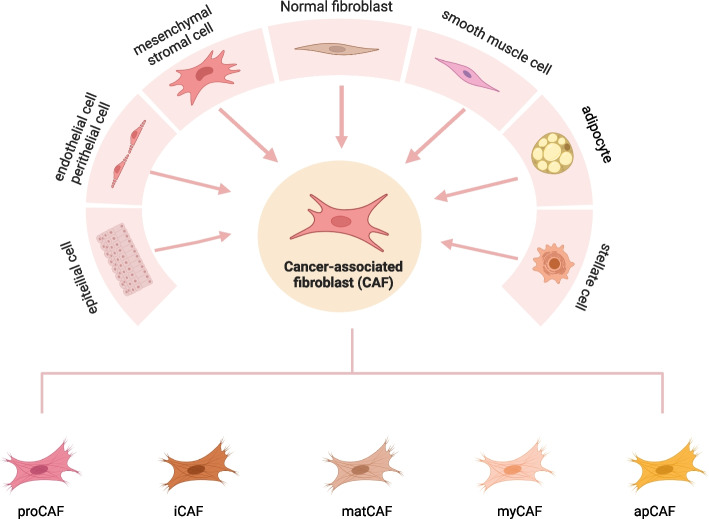
Origin and classification of cancer-associated fibroblasts. This schematic highlights the diversity of the origins and subtypes of cancer-associated fibroblasts (CAFs). ProCAF, progenitor of cell cancer fibroblasts; apCAF, antigen-presenting tumor-associated fibroblasts; myCAF, myofibroblasts; iCAF inflammatory fibroblasts; matCAF, matriculogenesis-associated fibroblasts

However, the origin of CAFs in breast cancer is relatively homogeneous. Julia M. Houthuijzen et al. demonstrated conclusively that CAFs in breast cancer stem predominantly from NFs, employing complementary transplantation of multiple genetically engineered mouse models(GEMMs). Furthermore, they observed that CD26^+^ NFs may serve as precursors for iCAFs and suggested the possibility of CD26^-^ NFs converting into myCAFs [26]. The diversity of CAF subtypes has become increasingly apparent through comparisons of their differential molecular expression, currently categorized into eight clusters in breast cancer and four in lung cancer. Lena Cords analyzed the distribution and function of CAFs in lung non-small cell carcinomas, utilizing 618 samples with a 15-year follow-up period. The study revealed that the specific type of CAFs significantly influences the prognosis of these tumors [27]. Regarding CRC, Ambre Gigley has discerned two distinct subtypes of CAFs from a meticulous analysis of six CRC liver metastasis samples. Intriguingly, she uncovered a functional parallelism between these CAF subtypes and those observed in other solid tumor types, emphasizing the potential significance of this discovery across a broader cancer context [28]. Later, antigen presentation-associated fibroblasts(apCAFs), metabolic cancer-associated fibroblasts (meCAFs), and complement-secreting fibroblasts(csCAFs) were progressively uncovered. Furthermore, identifying human pancreatic CAFs through scRNA-seq technology, coupled with the analysis of surface markers, unveiled a minute population of α-SMA/p-STAT double-positive cells, affirming the hypothesis that additional subtypes may exist [29]. Recently, Bonan Chen established a universally applicable molecular typing system for CAFs on a single-cell pan-cancer scale, utilizing 12 distinct solid tumor types and concluded that the phenotypic turnover of CAFs is continuously variable and occurs sequentially at different stages of tumor development (Fig. 1) [30].

The intricacy of CAFs is reflected across various organs, and their origins, classifications, functionalities vary within distinct regions of the same organ, potentially undergoing modifications in response to alterations in the surrounding environment. The lack of definitive CAF markers complicates isolating and purifying these cells, and the need for specific markers of CAFs has hindered experimental research on their functions. However, experimental comparisons have conclusively demonstrated that CAFs in various cancer types of distinct organs exhibit overlapping gene expression and functionality patterns. Consequently, whether this shared expression and functionality contribute to the metastasis of cancers across organs remains a focal point for future research endeavors.

### Heterogeneity of functions

The functional diversity of CAFs illuminated through single-cell RNA sequencing, multiple immunostaining techniques, and diverse genetic mouse models [31]. This encompasses but is expansive beyond facets such as remodeling the cancer stroma, angiogenesis, metastasis, immunomodulation, metabolic reprogramming, and treatment resistance.

myCAFs dominate immunosuppression, ECM deposition, metabolic reprogramming, drug resistance, metastasis, and other pro-cancer effects in pancreatic and breast cancers [32]. In LC myCAFs induce brain metastasis through the MET-HGF pathway and excrete substantial quantities of MMP11 and remodeling factors, which modify the ECM to hinder drug delivery, ultimately resulting in a dismal prognosis [24, 33]. Non-small cell lung cancer (NSCLC) is no exception, as mir-55, which is secreted by myCAFs, plays a significant role in conferring resistance to cisplatin [34].

iCAFs are frequently perceived as inflammatory agents in solid tumors, eliciting immunosuppression and enhancing metastasis alongside the supportive influence of cytokines [26, 35, 36]. Recently, it has been discovered that iCAFs, mediated by IL-1, are also implicated in the adverse effects of radiotherapy [37]. Although it is widely acknowledged that iCAFs function as tumor-promotive cells, they may also serve as indirect indicators of sustained immune reactions, stemming from their robust expression of interferon(IFN)-induced chemokines such as CXCL9, CXCL10, and CXCL11, and given the complexity of the IFN, tumor-promoting and anti-tumor properties of iCAFs cannot be determined yet [38]. Meanwhile, the congregation of iCAFs in the tumors of patients who did not relapse after chemotherapy suggests that iCAFs perform a specific tumor-suppressive role [39]. In 2019, Elyada and his team discerned novel subtypes of CAFs within PDAC that stimulate CD4^+^ T cells in an antigen-presenting capacity [40]. Additionally, csCAFs, possessing potential bidirectional influences on tumor progression, enhance inflammatory and immune responses within the TME through the expression of complement regulators [31, 32, 41].

Due to the diverse functions of CAF subtypes, it is more important to identify the subtypes of CAFs for precise targeting. However, the need for specific markers for CAFs has hindered experimental research on CAFs. Notably, New extraction methods suggest that different types of CAFs can be isolated by comparing the expression of vital functional signals when co-cultured with cancer cells. The fact that CAFs are phenotypically and functionally unstable cells whose functions may be disturbed by TME changes highlights the importance of signaling crosstalk between cells, and the study of the crosstalk mechanism will help induce the anticancer function of CAFs (Table 1).

**Table 1:** Types and functions of cancer associated fibroblasts(CAFs)

Type of Cancer	Subtype	CAF cluster	Marker	Function	Reference
Pancreatic cancer	proCAF	-	NFIX	(1) origin of CAF (2) regulates cell proliferation	[1, 2]
	iCAF	-	IRF9 CXCL12 CCL2 IL-6	(1) regulate immune (2) promote tumor (3) suppress tumor	[29, 42, 43]
	matCAF	-	CREB3L1	(1) ECM remodel (2) poor prognosis	[29, 44]
	myCAF	-	MEF2C ɑ-SMA FAP	(1) contractions (2) angiogenesis (3) drug resistance (4) poor prognosis	[29, 44]
	apCAF	-	MHC II CD74	immunosuppression	[45]
Breast cancer	iCAF	IL-iCAF		promote inflammation	[46]
		detox-iCAF		detoxification	[46]
		INFϒ-iCAF	CD47	-	[46]
	myCAF	ecm-myCAF	LRRC15	(1) ECM remodel (2) Immunotherapy resistance	[46, 47]
		TGFβ-myCAF	-	Immunotherapy resistance	[46]
		wound-myCAF	-	immune protection	[46]
		IFNɑβ-myCAF	-	Immunotherapy resistance	[46]
		acto-myCAF	-	-	[46]
Colorectal cancer	ECM-C AF	CP-CAF	FAP PDGFRA LTBP2	(1) angiogenesis (2) ECM remodel	[34]
		CS-CAF		(1) secrete complement (2) immunosuppression	
	Ctr-CA F	Ctr-CAF-I	RGS5 MCAM MYH11	contractions	
		Ctr-CAF-II			
Lung cancer	iCAF	iCAF	CXCL12 CXCL14	(1) Immunosuppression (2) drug resistance	[17, 32]
		iCAF-2	CCL-2 IL-6	immunosuppression	[17, 32]
	myCAF	-	ɑ-SMA MEF2C	brain metastasis angiogenesis	[48]
	apCAF	-	STC-1 VEGFA PDGFC	bone metastasis angiogenesis	[32, 49]

## The tumorigenicity of CAF by interaction in TME

TME is characterized by an abundance of immune cells, extracellular matrix, cancer cells, and CAFs. CAFs, as pivotal element within TME, engage in a multitude of interactions with other TME components. They secrete cytokines, enzymes, and other substances to facilitate inter-component interactions through signaling pathways. Furthermore, in response to the challenging conditions present within TME, CAFs also reprogram cancer cells through metabolites, thereby promoting persistent cancer growth.

### Crosstalk between CAFs and immune cells

In normal tissues, the immune system inhibits cancer through mechanisms such as immune clearance, immune surveillance, and immune defense. In tumor tissues, the immune system is suppressed by various cytokines, chemokines, and metabolic products, resulting in the polarization of immune cells towards a cancer-promoting direction and facilitating immune escape by the tumor. CAFs play a critical role in this process. Although studies have shown that CAFs exert both immunosuppressive and immune-promoting effects in TME, CAFs are more inclined to exert immunosuppressive effects [50, 51]. CAFs affect not only innate immune cells but also adaptive immune cells.

#### CAFs and innate immunity

##### CAFs and TAMs

Tumor-associated macrophages (TAMs), the significant immune cell population within the TME, are categorized into M1 and M2 subtypes. The M2 subtype specifically secretes CCL22, a chemokine that attracts regulatory T (Treg) cells. These Treg cells, in turn, suppress the anti-tumor immune response of T cells, fostering tumor progression and contributing to an unfavorable prognosis for patients. Additionally, the M2 subtype promotes angiogenesis and tumor invasion, further exacerbating the detrimental effects on patient outcomes [52–55]. In 2016, Engler AJ and his colleagues discovered that fibroblast activation protein-α(FAP), expressed by CAFs, modifies collagen fibers within the ECM and facilitates macrophage adhesion to these fibers through the class A scavenger receptor(SR-A/CD204). This process culminates in an association with the M2 phenotype, elucidating a mechanism of communication between fibroblasts and macrophages that operates through the FAP-ECM-SR-A axis [56]. Subsequently, a large number of cytokines have been extensively explored. It is suggested that the secretion of pro-inflammatory cytokines IL-1, IL-6, IL-8, IL-10, TGF-β, and chemokines C-X-C chemokine ligand (CXCL)-1 and CXCL-2 by CAFs could promote monocyte recruitment and the differentiation of cancer-associated macrophages from M1 to M2 [31, 57, 58]. Concurrently, TAMs modulate the activation of CAFs through the release of CXCL12 and IL-6 [59]. Through rigorous in vitro co-culturing experiments, TAMs induced N-cadherin, α-SMA, FAP, and vimentin in human mesenchymal stem cells (MSCs). This evidence underscores the profound impact of macrophages in facilitating the development of CAF-like traits within MSCs, offering further insights into the intricate interplay and mutual influence between these two cell types [60]. Recently, Raymant M uncovered a significant aspect of pancreatic cancer liver metastasis, highlighting the importance of macrophages in activating CAFs. Specifically, TAM-derived pro-granule proteins activate myCAFs in liver metastases through JAK/STAT3 pathway activation. Once activated, myCAFs secrete osteopontin, which counteracts immunosuppression by macrophages and CD8+ T cells, thus promoting the liver metastatic process of pancreatic cancer [61]. Surprisingly, TGF-β secretion by tissue damage-associated macrophages in non-malignant diseases promotes fibroblasts differentiation and collagen secretion, ultimately contributing to scarring. However, the question remains: Is this phenomenon intricately linked to the genesis of malignant tumors? The answer to this enigmatic query remains to be unearthed through further scientific exploration [62, 63].

Despite ample research indicating that TAMs and CAFs mutually reinforce their cancer-promoting functions, the intricate mechanisms underlying their interactions remain elusive. Further refinement is necessary to ascertain whether distinct pathways of their interplay exhibit cross-talk and whether these cells possess alternative modes of communication, in addition to cytokines.

##### CAFs and TANs

Neutrophils also play indispensable roles in innate immune cells. The neutrophils inhabiting TME are aptly designated tumor-associated neutrophils (TANs). Akin to TAMs, TANs can be categorized into two distinct types: N1 and N2. Among them, N1 is considered a key player in anti-tumor function, while N2 is perceived to contribute extensively to a tumor-supportive role, including the initiation and propagation of cancer cells, their angiogenic potential, and their tendency toward infiltration and metastasis [64]. There is also a positive feedback loop between CAFs and TANs, but the specific mechanism remains unclear due to the lack of relevant research. Currently, clinical specimens show that CAFs amplify N2-polarized TANs via upregulating the CLCF1-CXCL6/TGF-β signaling axis in hepatocellular carcinoma (HCC) [65]. At the same time, CAFs generate amyloid-β, a substance that triggers neutrophils to emit histone-conjugated nuclear DNA and cytotoxic granules in the form of extracellular traps (NETs). These NETs have been established as factors that promote inflammation and metastasis. In turn, NETs can further stimulate CAFs, leading to the induction of the myCAFs phenotype, thereby augmenting the probability of liver micrometastasis in PDAC [66, 67]. The information about the crosstalk between CAFs and TANs remains scarce. Despite a mutual positive feedback loop between these two entities, the precise underlying mechanism remains elusive, necessitating further exploration into the role TANs play in tumor progression.

Beyond their primary function of eradicating tumor cells, immune cells occupy a significant position in the secretion of inflammatory mediators. The investigation into how they induce phenotypic changes in CAFs and the consequences of these changes on the survival conditions of CAFs forms an exciting frontier in unraveling the evolution of the tumor microenvironment.

##### CAFs and other innate immune cells

Mast cells (MCs) exhibit complex dual roles in tumors. On the one hand, they contribute to tumor promotion by secreting matrix metalloproteinase-9(MMP-9) and vascular endothelial growth factor(VEGF). On the other hand, MCs inhibit tumor invasion and growth through the secretion of IL-1 and IL-6 [53]. Certainly, the release of IL-13 and tryptase by MCs triggers significant proliferation of CAFs. Increased CAFs subsequently lead to TME fibrosis and suppress anti-tumor immunity [68]. Other than that, an intricate interplay exists between natural killer (NK) cells and CAFs in innate immunity. One important molecule involved is TGF-β, although the specific mechanism remains unclear. CAFs inhibit NK cells function in both direct and indirect ways, secreting PGE_2_ to induce the conversion of NK cells to an inactivated phenotype and modulating NK cells activation-related receptors on tumor cells. Interestingly, NK cells can promote CAF-induced inhibition loop formation by enhancing the secretion of PGE_2_ [69].

#### CAFs and adaptive immune cells

Solid tumors can be classified as “hot” and “cold” tumors according to immune status, and pancreatic cancer is classified as “cold” tumors owing to immune exlusion [70]. In PDAC, CAFs prevent effective adaptive immunity mainly by preventing T cell recruitment, activity, proliferation and promoting polarization of T2 subtypes. On the one hand, CAFs secrete CXCL12 to cover tumor cells to prevent CD8^+^ T cells from aggregating near the tumor [71]. In CRC, CAFs, owing to their expression of PD-L1/2 molecules, suppress the proliferative response of activated CD4^+^T cells by impeding IL-2 production or elicit immunosuppression through the secretion of CXCL5, which subsequently activates the PI3K/AKT pathway to induce PD-L1 expression in cancer cells [72, 73]. Nevertheless, clinical research on the downstream mechanisms by which CAFs inhibit T cells via immune checkpoints, and therapeutic agents specifically targeting PD-L1/2 remains scarce. On the other hand, exosomes derived from CAFs upregulate PD-L1 expression in cancer cells, thereby enhancing the apoptotic rate of T cells and impairing their proliferation capabilities [22, 74–77]. Recently, Varveri et al. found that CAFs suppress immunity via synapses with Tregs, dependent on autophagy. Moreover, the latest research shows that blocking the IL-6 factor produced by CAFs through autophagy favors anti-tumor immunity over immunotherapy [78, 79]. CAFs are plastic, and alterations can influence their phenotypic expressions in their surrounding environment. Felix Simon Ruben Picard and colleagues discovered an unconventional CD8^+^T cell subset (Tc17) that produces IL-17A, a cytokine that converts CAFs with IL-17 receptors into iCAFs. In turn, Tc17-iCAFs support the differentiation of T cells towards the Tc17 phenotype and promote tumor growth relentlessly through the secretion of IL-6 [80]. Meanwhile, other CAF subtypes, such as apCAF, indirectly inhibit the proliferation of CD8^+^T cells by inducing Treg cells or directly trigger the apoptotic mechanism of T cells to suppress immunity [31]. In conclusion, Tregs are essential mediators in the mutual regulation of CAFs and adaptive immunity; exploring the mechanisms by which both CAFs and Tregs interact has the potential to improve immunity, and explorations on the biological behaviors of CAFs will be beneficial in refining the diversity of mechanisms of mutual communication.

The immune system is not isolated from each other but is an interactive system. There is also interference between innate immunity and adaptive immunity. Increased activating STAT3 activity in macrophages leads to impaired T-cell responses and limits T-cell function, thereby inducing PD-L1 expression in neutrophils [43, 81]. Moreover, the critical role of STAT3 activation in CAFs inducing MDSC differentiation and mediating immunosuppression was found in hepatocellular carcinoma with PDAC [82–84]. Hence, targeting STAT3 signaling can improve immune efficacy. Furthermore, CAF-induced macrophages can also inhibit the killing and activation of NK cells [85].

**Fig. 2:**
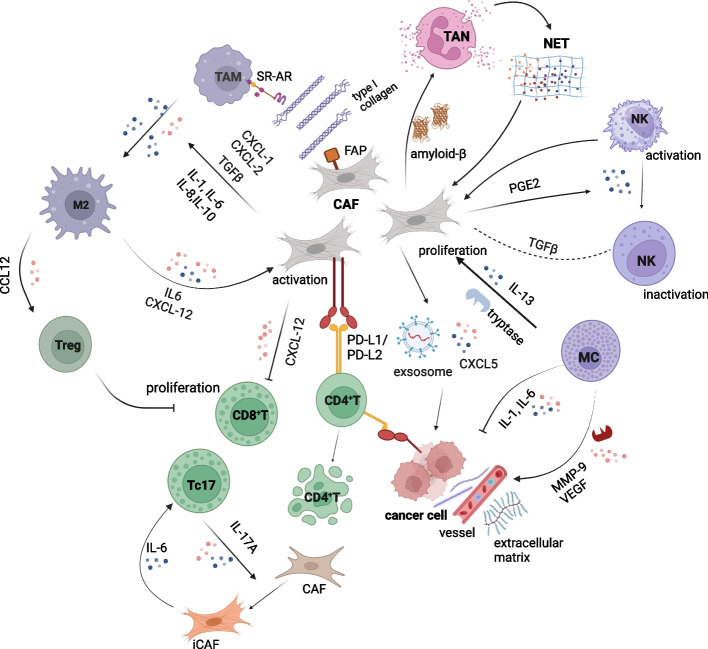
Crosstalk between cancer associated fibroblasts and immune cells. Cancer associated fibroblasts (CAFs) play a crucial immunosuppressive role in the tumor microenvironment. By secreting C-X-C chemokine ligand(CXCL)1, CXCL2, CXC12, Interleukin(IL)-1, IL-6, IL-8, Il-10, transforming growth factor(TGF)-β, C-C chemokine ligand 12(CCL12), prostaglandin E_2_(PGE_2_), CAFs regulate immunity by promoting the conversion of macrophages (TAMs) to a pro-cancer phenotype, inactivating natural killer (NK) cells, and preventing the aggregation of cytotoxic T (CD8^+^T) cells. Certainly, immune cells such as TAMs, NK cells, CD8+T cells, mast cell (MCs), and immunomodulatory T cells (Tregs) can secret IL6, IL13, CXCL12, trypsin, TGF-β to activate CAFs and promote their proliferation and differentiation. Furthermore, CAFs mediate the biological behavior of TAMs related to fibroblast activation protein (FAP) and scavenger receptor (SR-A/CD204) on TAMs. CAFs also induce apoptosis of CD4^+^T cells by up-regulating the autoimmunosuppressive checkpoint molecules programmed cell death protein 1 and 2(PD-L1/PD-L2) and their secretion of amyloid-β protein prompts neutrophils (TANs) to secrete extracellular traps (NETs) for feedback activation of CAFs

Overall, macrophages serve as a pivotal connector between diverse immune cells and assist CAFs in promoting tumor cell growth. Among the various crosstalk, there are two strategies to modulate tumor development: one is to block the activation of CAF subtypes that suppress immunity, and the other is to disrupt the connection between CAFs and immune cells. Among these, the role of TGF-β and inflammatory factors is not negligible [86]. Notably, the signaling pathways involving TGF-β and IL-1 are imperative for the activation and intricate crosstalk of CAFs. Furthermore, IL-6 derived from CAFs emerges as a significant contributor to the immune evasion mechanism observed in PDAC, highlighting its critical involvement in disease progression [87]. In instances, when precise targeting of CAFs presents a challenge, we can interfere with the signaling cascade that enables its immunosuppressive capabilities, thereby dismantling the immune evasion niche established by tumor cells. Current research has elucidated the effects of CAFs on the immune system, but there is limited information about the immune system's counteracting effects on CAFs. Addressing this gap could lead to therapeutic breakthroughs if further understanding is achieved (Fig. 2).

### Crosstalk between CAFs and ECM

Extracellular matrix (ECM) imbalance in tumors involves four aspects: ECM deposition, post-translational chemical modification, ECM degradation, and tissue force-mediated physical alterations [88]. Unbalanced ECM, characterized by densely cross-linked stromal fibers, actively activates tumor cell signaling, expediting the fibrotic response. This, in turn, diminishes the adhesion of tumor cells to the ECM, disrupts cell polarity, and triggers a cascade of tumor-invasive biochemical pathways, ultimately fostering tumor metastasis [89–98]. Especially in pancreatic cancer, a tumor with 50%-80% fibrous stroma, the role of ECM cannot be ignored [99–101].

**Fig. 3:**
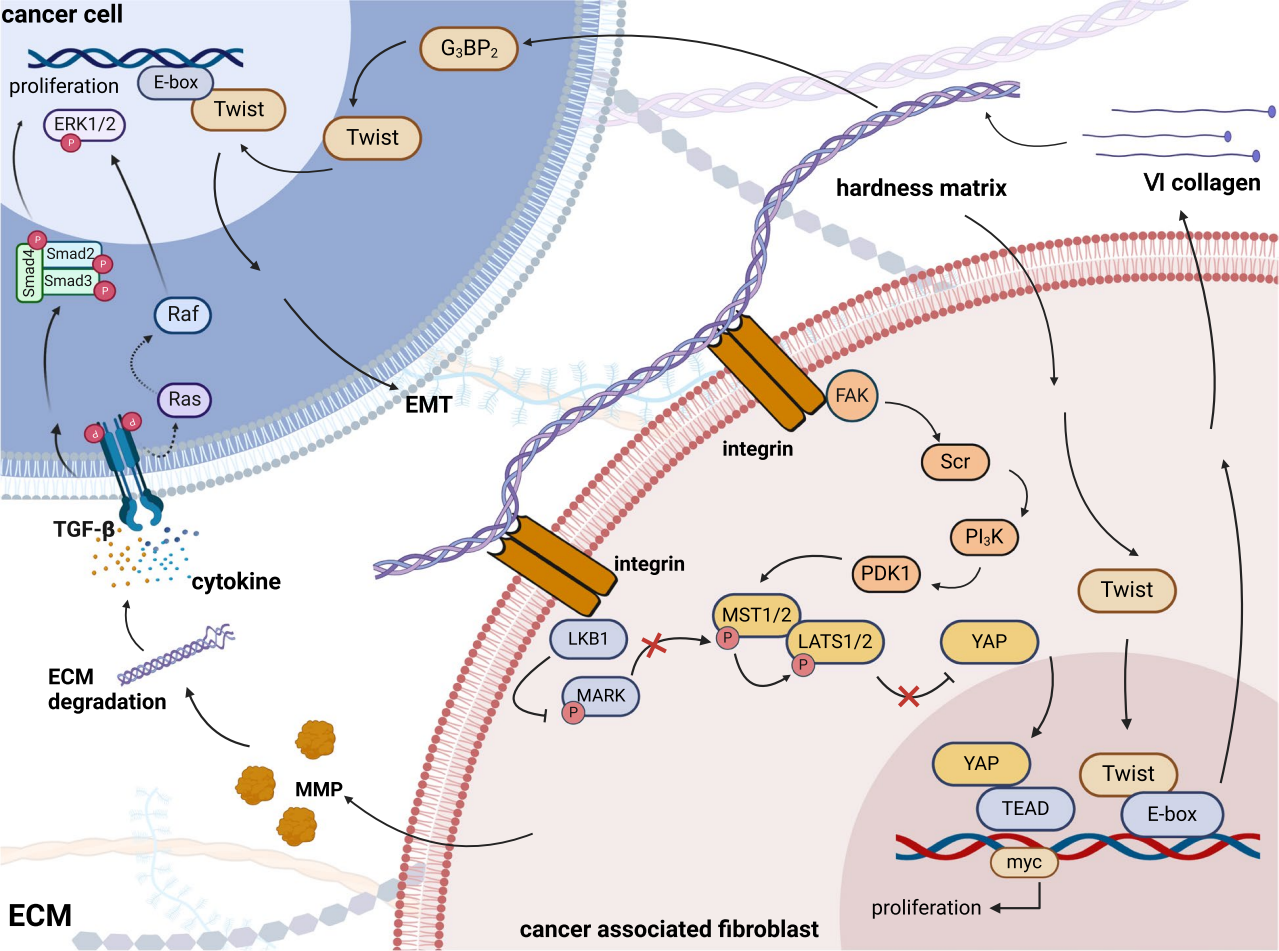
Cancer-associated fibroblasts crosstalk with extracellular matrix.Cancer-associated fibroblasts (CAFs) affect the deposition and degradation of extracellular matrix (ECM) and promote tumor growth by affecting the ECM. CAFs promote the degradation of the ECM through the secretion of metalloproteinases (MMPs) leading to the release of various growth factors from the ECM to activate the growth signaling pathway of cancer cells. At the same time, the changes in ECM counteract the CAFs and tumor cells. The main manifestations are: (a) the hard ECM triggers the release of Twist from CAFs, prompting CAFs to secrete type VI collagen fibers single chain, which exacerbates the hardness of ECM. (b) the hard ECM triggers Twist in cancer cells, which triggers epithelial-mesenchymal transition (EMT) and promotes metastasis. In addition, collagen fibers of ECM inhibited the Hippo pathway by connecting with integrins of CAFs, and up-regulated YAP expression to activate growth signaling and promote cell proliferation

CAFs are the significant contributors to ECM deposition. Tumor cells and CAFs jointly secrete lysyl hydroxylase(LH) in conjunction with matrix metalloproteinase-9(MMP-9), which activates the TGF-β signaling pathway and promotes collagen cross-linking within the stromal matrix, subsequently enhancing tumor stiffness [102–105]. The rigid matrix elicits mechanical signals that influence diverse cellular responses, fostering pro-cancerous biological activities and serving as a physical shield, protecting malignant cells from the effects of chemotherapeutic agents [106–108]. Specifically, ECM fosters the aggregation of integrins [88], which subsequently stimulates the activity of the transcription factor YAP in CAFs. This activation occurs via the negative modulation of Hippo signaling by LKB1 [109], FAK/Src/ PI_3_K [110] and JNK [111]. Ultimately, YAP binds with TEAD, thereby enhancing myc transcription and promoting proliferation [112], and enhances the adhesion of metastatic malignant cells to the tumor endothelium to promote cell migration (Fig. 3) [113]. Despite numerous experiments geared towards identifying the upstream regulators of the Hippo/YAP pathway, the precise upstream regulatory mechanisms remain elusive. The intricate interplay between various signaling pathways continues to pose challenges, and whether the classical RAS pathway impacts the upstream of the Hippo pathway remains unanswered. In breast cancer, it was found that the high stiffness of the stroma directly anchors G_3_BP_2_ in the cytoplasm to promote Twist1 release and nuclear translocation, which triggers epithelial-mesenchymal transition (EMT) of the cells to promote metastasis. Surprisingly, Twist1 can directly stimulate the expression of type VI collagen α1 chain within CAFs, subsequently activating CAFs and intensifying ECM stiffness, thereby perpetuating a vicious cycle [114, 115]. Twist has the potential to be a future therapeutic target based on its broad role in cancer development (Fig. 3). Other studies have demonstrated that the inflammation or injury prompts fibroblasts to synthesize and deposit ECM proteins, generating temporary matrix and mechanical stress that induces NFs differentiation towards myCAFs. Moreover, NFs generate fibronectin EDA splicing variants under the influence of TGF-β, thereby increasing cellular tension, recruiting ɑ-SMA+ myCAFs, and raising the likelihood of tumorigenesis [116–119].

The ECM constitutes a highly dynamic architectural framework, and amidst its remodeling process, CAFs emit enzymes that concurrently facilitate matrix cross-linking and stimulate catabolism. The presence of CAFs in tumor cells, which secrete LH in conjunction with MMP-9, significantly enhances the likelihood of collagen fiber degradation and further fosters cross-linking in the milieu of TGF-β [24, 102–105, 120, 121]. The remodeled ECM, in turn, increases interstitial fluid pressure in the tissue and activates CAFs to upregulate TGF-β and MMPs [122, 123]. MMPs, as extensively studied matrix-associated enzymes, are found in increased content in tumor tissues [123], suggesting that proteases are actively involved in ECM remodeling assisting in tumor progression, similarly to stromal enzymes such as lysyl oxidase(LOX), which increases ECM stiffness by binding collagen filaments and cross-linking collagen networks [124–127]. With the degradation of ECM, the dense mesh structure will create an exit for cancer cells shuttling and release many growth factors, chemokine activation. When degraded, CAFs change the collagen fiber orientation through physical action, removes the metastatic obstruction, and promotes the travel of cancer cells ECM [128]. Therefore, inhibiting matrix-associated degradation enzymes or disrupting the interaction between CAFs and ECM may delay ECM degradation. However, preventing ECM degradation alone may not yield the desired therapeutic outcomes. Some studies have shown that degraded small fragments of ECM may have antitumor effects. Cheng Y et al. also found that degrading the extracellular matrix can increase drug permeability and enhance tumor ablation [129, 130].

Although hyaluronic acid therapy has emerged as the primary therapeutic approach for targeting ECM by dissolving collagen fibers, it is crucial not to perceive ECM as a standalone fibrous component but rather as a delicate equilibrium between degradation and synthesis. The regulatory role of CAFs and cancer cells in this process should be emphasized throughout the dynamic changes, and attempts should be made to find regulatory pathways to intervene. Experimentally determining a range of ECM hardness that hinders cancer growth and consequently guiding the administration of drugs could avoid the drawbacks associated with blindly eliminating the ECM, which brings about an opportunity to restore the balance of the TME.

### Crosstalk between CAFs and cancer cells

#### Interaction between CAFs and tumor cells in inflammatory TME

**Fig. 4:**
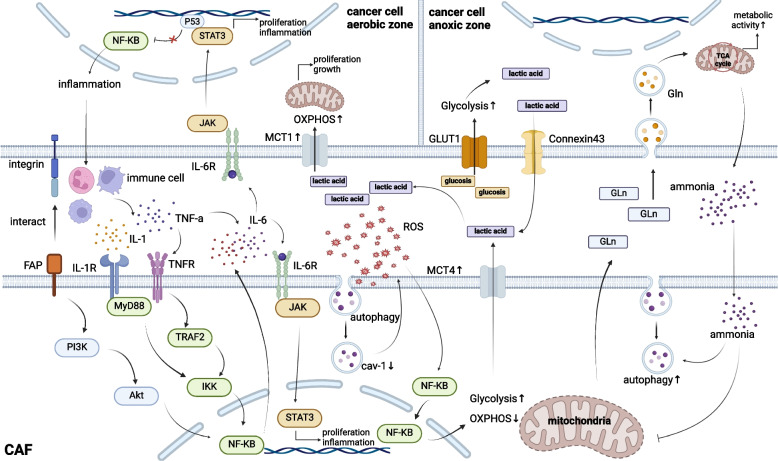
Cancer-associated fibroblasts crosstalk with cancer cells under inflammation and stress. In the inflammatory state, cancer cells interact with cancer-associated fibroblasts (CAFs) in two ways: (a) Abnormally active NF-KB of cancer cells with P53 mutation stimulates immune cells to secrete tumor necrosis factor (TNF-a) and IL-1, and inflammatory factors activate the NF-KB of CAFs to further release the inflammatory factor IL-6. With the auxiliary effect of TNF-a, IL-6 is taken up by the cancer cells and CAFs uptake to activate STAT3 to promote proliferation. (b) CAFs activate the NF-KB activity of CAFs through direct contact between fibroblast activation protein (FAP) and cancer cell integrins, which secretes a large amount of inflammatory factors to exacerbate the inflammatory state, forming a positive feedback. Under stress, CAFs interact metabolically with cancer cells. (a) glucose metabolism: under oxidative stress, CAFs undergo autophagy and downregulate Caveolin-1 (Cav-1), loss of Cav-1 exacerbates oxidative stress.Oxidative stress activates NF-kB to inhibit oxidative phosphorylation (OXPHOS) and enhances glycolysis to produce lactic acid, which is absorbed by cancer cells in the aerobic zone and enhances OXPHOS to accelerate the growth and save a large amount of glucose. Cancer cells in the hypoxic zone absorbed glucose and produced lactic acid by glycolysis to supply cancer cells in the aerobic zone to meet their energy demand. (b) Protein metabolism: cancer cells autophagically absorb exogenous glutamine (Gln) to produce large amounts of ammonia through the tricarboxylic acid cycle (TAC), which inhibits the mitochondrial function of CAFs and strengthens autophagy; and then CAFs reduce the utilization of their own Gln and release it to supply the uptake of the cancer cells to activates the mitochondrial activity of the cancer cells as well as accelerate metabolism

The inflammatory environment is a common microenvironmental state in tumor tissues, centered on critical inflammatory pathways such as STAT3, NF-kB, which facilitate interaction between CAFs and tumor cells. During the initial stages of the tumor, resident immune cells within the tissue microenvironment secrete the pro-inflammatory cytokine IL-1β, serving as a primary activator of NF-κB to initiate crosstalk between CAFs and tumor cells [131]. In cancers, CAFs cause aberrant activation of NF-κB in the form of pro-inflammatory factors or direct contact with cancer cells [132], and the derived cox-2 factor stimulates paracrine secretion from neighboring cancer epithelial cells to the point of angiogenesis, tumorigenesis [133]. It has been observed that P53-mutated cancer cells exhibit active NF-κB expression, prompting the secretion of TNF-α by target cells. TNF-α, in turn, stimulates the upregulation of NF-κB in CAFs, enhancing pro-inflammatory factors and thereby exacerbating inflammation [134, 135]. Additionally, TNF-α potentiates the activity of IL-6, which activates STAT3 expression in cancer cells, enhancing the self-renewal and invasive capabilities of pancreatic cancer stem cells [136–138]. Furthermore, it indirectly modulates the paracrine secretion of CAFs via the SHH pathway, exacerbating stromal fibrosis and fostering a tumor-permissive microenvironment conducive to growth and drug resistance (Fig. 4) [132]. Notably, fueled by the inflammatory milieu, the interplay between CAFs and cancer cells becomes significantly more vigorous, profoundly influencing cancer progression. The pivotal role of CAFs in the inflammatory transformation of cancer merits heightened emphasis in forthcoming research endeavors.

#### Metabolic crosstalk between CAFs and cancer cells in response to stress

Cancer cells and their surrounding stromal counterparts suffer from a deprivation of oxygen and nutrients, encased within a compact collagen matrix. This confinement, coupled with intrinsic genetic alterations and external stressful conditions, collaboratively reconfigures their metabolic pathways in a bid for survival [139–141]. The earliest known metabolic model was the “Warburg Effect” proposed by Otto H. Warburg in the early 1920s [142]. Recently, Pavlides et al. proposed the “reverse Warburg effect” to highlight that cancer cells with mitochondrial function are metabolically interdependent with CAFs to produce more energy [143]. Subsequent studies have shown that co-culturing pancreatic cancer cells with CAFs increases the expression of critical enzymes involved in lactate import and the tricarboxylic acid(TCA) cycle, leading to the metabolic coupling between tumor cells and CAFs [144]. The initial step driving the metabolic coupling between CAFs and tumor cells involves the tumor cells inducing oxidative stress in CAFs. Although the exact mechanism of this induction is not yet fully understood, studies have shown that the level of cellular stress response correlates with the proximity of the stromal cells to the tumor. Specifically, CAFs located closer to the tumor exhibit higher levels of stress response, which in turn induces oxidative stress in the cancer cells [141, 145]. It is worth noting that various physiological responses to external stimuli are not solely detrimental but complex and diverse. In the initial phases of neoplastic progression, the stress response can inhibit tumor progression and protect cellular integrity. However, as the tumor progresses, chronic and persistent oxidative stress leads to disorders in TME and metabolic abnormalities [141]. Surprisingly, the pivotal regulatory protein, nuclear factor NRF2, occupies a significant position in the oxidative stress response. One of the key regulators of NRF2 is Caveolin-1(Cav-1), which has garnered much attention recently. Martinez-Outschoorn UE research has identified the deletion of Cav-1 as a defining characteristic in response to oxidative stress in tumors, further underscoring its indispensability for CAFs to establish a metabolic parasitic interaction with tumor cells. At the molecular level, oxidative stress mediates the activation of HIF-1 and NF-κB in CAFs, further inducing autophagic degradation of Cav-1, and Cav-1-deficient CAFs exhibit protection against apoptosis in cancer cells [146]. Thus, Cav-1 has also become an indicator of recurrence and prognosis in breast cancer [147]. However, high expression of Cav-1 in CAFs in the stroma also brings about pro-invasive effects and high expression of Cav-1 has been found in breast and colon cancers, where it has been found to regulate Rho-mediated cellular contraction through p190-dependent regulation leading to structural disorganization of the ECM, and enhancement of peripheral cell migration [148]. Because cancer arises from a malignant phenotypic transformation triggered by oncogenic factors inducing DNA damage in normal tissues, which accumulate genetic mutations, identifying the causes and mechanisms behind oxidative stress generation offers a potent approach to halt cancer development and progression.

CAFs experience metabolic reprogramming alongside tumor cells, attributed to their inherent high metabolic flexibility, intensified glycolytic activity, and diverse microenvironments within distinct compartments [149, 150]. When both the "Warburg effect" and the "Reverse Warburg effect" are established, the high expression of MCT1 in aerobic zone cancer cells took up lactate in the matrix as the substrate of OXPHOS. This metabolic process exacerbated oxidative stress, which activated HIF-1 and NF-κB in CAFs, increased glycolysis in CAFs, and reduced OXPHOS to produce a large amount of lactate. Subsequently, the upregulation of MCT4 expression on CAF cell membranes enhances the transport of lactate into the stroma, where it is then taken up by aerobic cancer cells, further propelling their proliferation [151–153]. Meanwhile, hypoxia triggers the activation of HIF-1 in cancer cells, leading to the upregulation of GLUT1, thereby enhancing glucose uptake [154, 155]. Subsequently, the glucose is converted into lactate via the heightened expression of intracellular lactate dehydrogenase A (LDHA) and the inhibition of pyruvate dehydrogenase (PDK). Ultimately, lactate is transported out of the cell by Connexin-43 and directed towards the oxygen-rich zone, which normal mitochondrial tumor cells uptake through the transport protein MCT1, then metabolized to alanine, glutamate [156]. Thus, more glucose is preserved for absorption by cancer cells in the hypoxic zone [157], enabling the nutrient metabolism to be recycled and efficiently harnessed within diverse zones of cancer tissues. This process intensifies the tumor's growth (Fig. 4). Studies have demonstrated that circulating lactic acid serves as a pivotal source of TCA intermediates, a crucial substance in addressing the shortage of nutrients essential for cellular nourishment. Furthermore, a decrease in pH within a high-lactate environment intensifies the intricate crosstalk metabolic pathways involving amino acids [158].

Autophagy frequently arises as a physiological mechanism where cells, failing to undergo apoptosis under pressure, temporarily preserve their energy metabolism through the breakdown of organelles and the generation of low-molecular-weight metabolites. This is viewed as a prompt reaction of tumors to escalating nutritional requirements [159, 160]. Recent research has demonstrated that autophagy plays a pivotal role in the crosstalk of protein metabolism between CAFs and cancer cells. Glutamine (Gln) significantly contributes to the cellular carbon source, supplementing glucose in this vital process [161]. It has been discovered that cells predominantly depend on exogenous Gln, metabolized by cancer cells to generate ammonia that is subsequently released into the stroma. This excessive ammonia stimulation of CAFs triggers a decline in mitochondrial activity, ultimately releasing even more Gln. This Gln is then sequestered by autophagy in cancer cells, augmenting their mitochondrial activity [162, 163]. In breast cancer, the high expression of myc in cancer cells promotes the secretion of exosomal microRNA-105, which acts on CAFs to stimulate myc activation to significantly increase the catabolism of Gln (Fig. 4) [164]. Yijuan Zhang and colleagues found that restricting glutamine increases in intracellular Ca2+, which activates CaMKK2-AMPK signaling and triggers AMPK-dependent macropinocytosis. This process provides essential amino acids from the extracellular environment to CAFs, thereby supporting cancer cells in a nutrient-deficient state [165]. Although the exact mechanism driving cytosolic Ca^2+^ increase upon L-Glutamine depletion is unclear, it may be related to endoplasmic reticulum stress [166]. The Gln-dependence of CAFs in breast cancer drives the migration of CAFs and cancer cells toward glutamine-rich regions in response to TRAF/AKT2 [167, 168]. Additionally, other amino acids are gradually being recognized for their roles. In PDAC, CAFs, primarily derived from pancreatic stellate cells (PSCs), support tumor metabolism through autophagy, similar to PSCs. Cristovão M. Sousa found that PSCs undergo autophagy upon stimulation by cancer cells and secrete non-essential amino acids such as alanine. This alanine is converted to pyruvate via the TCA cycle in cancer cells, facilitating the use of substrates like glucose to synthesize other molecules, thus enabling cancer cells to tolerate low-glycemic environments [169]. Therefore, it is worth considering whether the combination of pharmacological treatments that inhibit autophagy can avoid the side effects of conventional drugs and make the treatment more sensitive as a combined treatment option [170]. Regarding lipid metabolism, CAFs may facilitate lipid transport to cancer cells. In lung adenocarcinoma, colon cancer, and prostate cancer, CAFs have been shown to transport lipids into cancer cells via exosomes. However, the mechanism of this lipid transport in pancreatic cancer remains to be elucidated [170–172].

In conclusion, the metabolic crosstalk between CAFs and cancer cells is a crucial supportive mechanism in emergencies. Consequently, it is imperative to conduct further verification to ascertain whether applying radiochemotherapeutic treatments exacerbates the microenvironment's hypoxic or nutrient supply state promoting the metabolic crosstalk between CAFs and cancer cells.

## Cancer’s double-edged sword: CAFs

The functional complexity of proteins expressed by CAFs, the diversity of CAF isoforms, and their oncogenic and tumor-promoting roles render them a significant area of study. In highly fibrotic tumors such as pancreatic cancer, the presence of a large number of CAFs has an important role in treatment. Hence, interfering with the oncogenic function of CAF is of great clinical significance.

### Dual role of CAFs in cancer

While CAFs, as aforementioned, facilitate tumor growth, metastasis, and drug resistance via the secretion of cytokines, metabolites, or direct extensive crosstalk with components within the TME, Berna C. Özdemir et al. have surprisingly reported that the deletion of myCAFs not only fails to inhibit tumors but paradoxically augments their aggressiveness in pancreatic cancer [173]. By analyzing proteins with high expression of CAFs, it was discovered that tumor with reduced levels of α-SMA^+^ CAFs demonstrated a heightened tumor invasiveness, implying that CAFs expressing α-SMA+ have a tumor-limiting effect [31, 173]. Surprisingly, an in situ hybridization analysis of 71 human PDAC tissues revealed that individuals with Mefline-positive CAFs exhibited a more favorable prognosis. Conversely, CAFs devoid of Mefline expression showed inferior tumor tissue differentiation, aligning with the statistical analysis of human tissues. This finding implies that Meflin could potentially serve as a biomarker for anti-cancer CAFs (rCAFs), indicating its potential to hinder the progression of cancer [174]. Furthermore, it is postulated that the amalgamated distribution and contractility properties of CAFs have the potential to impede tumor progression by erecting a physical barrier that confines the expansion and motility of tumor cells [63]. While the anticancer attributes of CAFs have been recognized, a comprehensive understanding of the underlying anticancer mechanisms remains elusive. This limitation is partially attributed to the preferential selection of pro-tumorigenic phenotypic CAFs (pCAFs) by tumor cells, resulting in the gradual displacement of the anti-tumor phenotype during the early stages of phenotypic differentiation when CAFs are co-cultured with tumors. Consequently, the anti-tumor activity of CAFs is frequently overlooked. This underscores the necessity to refine experimental strategies by isolating and focusing on the tumor-promoting phenotype of CAFs at an early stage rather than competing with the anti-tumor phenotype [175].

### The significance of targeting CAFs

Currently, the primary treatment modalities for cancer encompass surgery, radiotherapy, and targeted therapy. In the realm of solid tumors, the objective of non-surgical treatments is to facilitate surgical interventions and amplify the efficacy of cancer eradication post-surgery. In adjuvant therapy, CAFs have an impact on efficacy. Radiotherapy, for instance, involves a non-discriminatory eradication of cancerous tissues, inherently resulting in inevitable side effects. Its singular focus on cancer cells overlooks the pivotal significance of the TME in cancer progression. By inducing circulating hypoxia, fostering vascular regeneration, inflammation, and fibrosis, alongside its cancer-killing prowess, radiotherapy paradoxically activates the intricate TME network, ultimately leading to the abnormal stimulation of CAFs, thereby fostering drug resistance or disease relapse. Hence, targeting CAFs in conjunction with radiotherapy is necessary to improve outcomes [176, 177]. Chemotherapy also triggers identical tissue, damage causing the activation of NFs, and the development of a CAF phenotype can facilitate cancer recurrence [178]. In addition, the effect of CAFs on the densification of extracellular matrix obstructs the penetration of chemotherapeutic drugs to a certain extent, and reasonable control of the amount and secretion function of CAFs can improve the effect of drugs. More importantly, CAFs have both anticancer and cancer-inhibitory effects. Precise extraction, targeting of cancer-promoting CAF phenotypes, and amplifying the function of cancer-inhibiting CAFs are important directions for future experimental and clinical studies.

### Challenges and opportunities for CAFs

Although a plethora of markers exist for CAFs, they suffer from a lack of specificity, rendering them indistinguishable from other cell types (Table 2). The significant heterogeneity of CAFs, coupled with the absence of definitive proteins, poses challenges in accurately identifying and isolating these cells. Moreover, in vitro studies have revealed that different CAF subtypes can be interconverted. The formation of iCAFs depends on the serial activation of IL-1/NF-ΚB/LIF/STAT signaling in CAFs, while TGF-β can antagonize this mechanism by downregulating IL-1R expression, promoting the phenotypic transition from iCAFs to myCAFs [17]. The phenotypic mobility of CAFs presents significant challenges in precisely tracking their specific phenotype. Furthermore, given the contractile nature of CAFs, meticulous modulation of contractility during tumor progression may determine the shift of CAFs from a tumor-restrictive phenotype to a pro-tumorigenic one [172]. Despite the inherent instability of the CAF phenotype, it presents a promising avenue to explore the potential transformation of their pro-cancerous nature into an anti-cancer phenotype. In the ongoing process of refining the classification of CAF subtypes [179], identifying distinct marker proteins specific to CAFs through comparing protein expression disparities between these two phenotypes and elucidating the mechanisms underlying phenotypic transformation will be paramount.

**Table 2:** Marker molecules for cancer associated fibroblasts(CAFs)

Type of CAF markers	Marker molecules	Reference
cytokine	TGFβ, VEGF, PDGF, EGF, FGF, PGE2, CTGF, SDF-1(CXCL12), WNTs	[180, 181]
membrane protein	PDGFRα/β, VCAM1, DDR2, TGFβRI/II, EGFR, FGFRs, BMPRI (BMPR1A/B)/BMPRII, podoplanin, FAP, Mefline	[10, 131, 173, 182]
Cytoplasmic proteins and skeleton	Desmin, FSP1/S100A4	[39] [51, 53]
ECM Components	collagen I, collagen II, fibronectin, tenascin C (TN-C), periostin LOX、 LOXL1, MMP, TIMP	[181, 183]

## Therapeutic strategies for CAFs

Three stages of CAF, from its origin, activation, and function, can be utilized as drug targets. The most direct therapeutic approach is to target the marker proteins of CAF. However, this is often challenging to achieve due to the lack of markers for CAF. Additionally, large amounts of cytokines are distributed in the extracellular matrix participating the entire process of CAF activation and cellular interactions, which represents an effective indirect method for targeting CAF. Therefore, we list the current therapeutic pathways for controlling CAF and their clinical trial phases.

### Targeting CAFs

There are two pathways for targeting CAFs: On the one hand, targeting CAFs for labeled protein therapy. The substantial stromal content of FAP contributes to an unfavorable prognosis, hence rendering treatment strategies against FAP as promising avenues for targeting distinct subtypes of CAFs, particularly in tumors exhibiting connective tissue hyperplasia. Such treatments have demonstrated commendable efficacy in enhancing the permeability of chemotherapeutic drugs [137, 138, 184–186]. Chimeric antigen receptor (CAR) engineered cell therapy has been a hot research topic in recent years. It has achieved significant efficacy, mainly in hematologic diseases, but remains challenging in solid tumors due to the lack of specifically targeted surface antigens. Wehrli M et al. used FAP with CD3 on T cells as an antigenic target constituting MesoFAP CAR-TEAM application to eradicate CAFs and cancer cells [187]. In addition, the combination of CD70-targeted drugs with cisplatin in non-small cell lung cancer has shown promising results in early clinical trials [188]. Van den Eynde has further validated CD70 as a promising antigenic target for IL-15-mediated CAR-NK cells in the treatment of solid tumors, positioning CD70-CAR-IL-15 NK cells as a promising therapeutic product poised for clinical trials [189]. The therapy for FAP encompasses the utilization of FAP inhibitors (FAPIs), which play a pivotal role in diagnostic imaging and targeted management of malignancies characterized by elevated levels of activated fibroblasts. Their minute molecular size, exceptional specificity, robust affinity, and advantageous pharmacokinetic properties render them suitable for this purpose. Presently, various FAPI compounds, including FAPI-02, FAPI-04, and FAPI-46, are undergoing rigorous clinical trials [190, 191].

On the other hand, inhibiting CAFs activity represents a novel therapeutic approach. Vitamins have been shown to have this effect; furthermore, the vitamin D receptor, as a transcription factor, regulates PSC cells to stop in a quiescent state, attenuating CAF activity [192]. All-trans retinoic acid (ATRA), based on the role of vitamin A in inhibiting PSC activation, also shows potential for treating pancreatic cancer [193]. Shi, b. et al. discovered that PDIC-OC triggers an endogenous reactive oxygen species(ROS) burst, leading to cytoskeletal dysfunction and apoptosis. This process inhibits TGF-β secretion and reprograms CAFs to transform CAFs into a quiescent state, thereby alleviating immunosuppression [194]. With advancements in nanotechnology, researchers and practitioners have harnessed its potential to develop groundbreaking treatment strategies. A comprehensive study conducted by Yaxian Li revealed that anti-PD-L1 & CXCR4 bispecific nanoantibodies effectively inhibit SDF-1 secretion by PSCs, thereby downregulating the PI3K/Akt signaling pathway to prevent CAFs activation [195]. Nanotechnology is effective not only for CAFs but also for ECM remodeling, which will be described below (Table .3).

**Table 3:** Targeted therapeutic drugs and functions

Target	Drug	Function	Reference
CAF	MesoFAP CAR-TEAM	CD3 molecules targeting FAP and T cells	[185]
	CD70-CAR-IL-15 NK	Antibody specifically binds CD70 on CAF	[187]
	FAPI-02、FAPI-04、FAPI-46	Inhibition ofFAP	[188, 189]
	ATRA	Induction of PSC quiescence	[191]
	PDIC-OC	CAF reprogramming	[192]
	anti-PD-L1 & CXCR4 bispecific nanoantibodies	Inhibition of CAF activation	[193]
TGF-β, TGF-βR	BiTP	Anti-PD-L1/TGF-β bispecific antibody	[196]
	SAR439459	Reduced TGFβ down-regulation of TGFβ pathway activation	[197]
	TGFβ vaccine	Reduction of TAM and CAF	[44]
	TGF-β1 Receptor Type I Inhibitor	TGF-β1 Receptor Type I Inhibitor	[197–199]
	LY3022859	Anti-TGFβreceptor type II monoclonal antibody	[200]
ECM	PEGPH20	HA degradation	[201]
	DAS@P/H/pp	HA degradation	[202]
	Lip-DTI/NO	HA and collagen degradation	[203]
	QUE	Delay ECM Remodeling	[204]

### Treatment of ECM

#### Targeted cytokines

CAFs convey messages in the TME by cytokines; thus, blocking such factors could be a novel strategy for cancer therapy. Experiments have demonstrated that TGF-β blockade increases tumor sensitivity to chemotherapeutic agents and shrinks the impact of CAFs [196]. Mace’s TA study found a positive correlation between TGF-β expression and PD-L1 expression, leading to the discovery of synergistic immune regulation [197]. Recently, Zhang H and his colleagues discovered that the anti-PD-L1/TGF-β bispecific antibody (BiTP) notably enhanced the alleviation of CAF-mediated immunosuppression. Furthermore, the potential utilization of this BiTP as a neoadjuvant therapy, in conjunction with chemotherapy prior to surgical intervention, may significantly increase the likelihood of successful surgical outcomes and improve patient prognoses [183]. Drugs that block the TGF-β receptor are also being heavily explored. For example, the initial clinical trial evaluating the anti-TGF-β monoclonal antibody SAR439459 in patients suffering from advanced solid tumors, which juxtaposed tumor biopsies against peripheral blood samples, observed consistent declines in TGF-β levels, thereby meeting the anticipated outcomes. However, due to the small sample size and lack of validated biomarkers, it was not determined that there would be sufficient therapeutic benefit in combination with cemiplimab [198]. Galunisertib (TGF-β1 receptor type I inhibitor) was tested in a phase 2 clinical trial with sorafenib in patients, achieving an acceptable safety profile and prolonging overall survival. Excitingly, as a combination therapy in patients with advanced rectal and lung cancers, the drug also improved complete remission rates and was well tolerated in clinical trials with a safety window based on the potential for cardiotoxicity [199, 200, 205, 206]. However, LY3022859 failed to achieve the phase I clinical trial goal due to the occurrence of infusion-related reactions in its application [206–208]. There are also related herbal ingredients used to treat PDAC, Wang et al. found that huacanin (HCS) can regulate the activity of the TGF-β/Smad pathway and control cancer growth rate [201]. Moreover, Perez-Penco M argues that a vaccine against TGF-β can effectively control the phenotype formation of myCAFs, reducing the proportion of TAM2 and favoring the restoration of the immune environment in the TME [209]. In conclusion, exploring the biological behavior and secretory factors of CAFs will provide additional pathways for treatment.

#### Treatment of ECM

The main components of ECM are collagen fibers and HA; thus, targeting both has become a significant strategy. Polyethylene glycol-recombined hyaluronidase (PEGPH20) can induce HA degradation [202], and initially remodel the TME under systemic application, thereby prolonging overall survival (OS); PEGPH20 has since been subjected to more extensive experiments. However, it has yet to be utilized due to its toxicity and side effects when combined with other drugs [139, 203]. To improve matrix densification issues, novel therapies have also emerged that carry a programmed nano-remodeling agent with hyaluronidase(Das@P/H/pp) [204]. A multifunctional liposome(Lip-DTI/NO) has been developed using photo-triggered cascade therapy in combination with nanotechnology to efficiently digest the collagen and HA and increase drug delivery [210]. A novel CAF-specific nanosystem (Dex-GP-DOCA, DPD) loaded with antifibrotic flavonoid compounds (Quercetin, QUE) has been developed by Xinyuan Zhou for their long-acting bioreactivity to FAP-α, delaying TME remodeling and enhancing clinical chemotherapeutic drugs [42]. Therefore, new opportunities will be provided for regulating the TME by utilizing nanotechnology to facilitate the entry and release of drugs into the dense matrix and reshape ECM (Table 3).

## Conclusion

CAFs in the stroma inhibit effective immunity and promote the transformation of immune cells to a cancer-promoting phenotype by secreting cytokines and chemokines. Activation of related signaling pathways and secretion of matrix-related enzymes enable deposition and decomposition of ECM, facilitating the transfer of malignant cells. Under various stress conditions, CAFs support cancer cells by activating signaling pathways and fueling their proliferation through metabolic reprogramming. A deeper understanding of the association of each component could provide broader avenues for treatment, such as TGFβ, IL-6, CXCL, and others, which are involved in multiple networks of relationships. Blocking their targets or reducing their production can be a supplementary method to alleviate tumor progression. In addition, more and more new methods such as nanotechnology, CAR-T, and vaccines are gaining attention. The ability to deliver drugs through diverse methods, extend the effectiveness of traditional medications, and achieve greater precision in targeting cells presents a welcome advancement. However, because of the complexity of the response in mice, its clinical applications require further testing. Moreover, based on our comprehension of the dual nature of CAFs and the paramount significance attributed to their effects, directing therapeutic strategies towards CAFs offers promising avenues for future cancer treatment. Nevertheless, a more exhaustive accumulation of experimental and clinical research evidence is imperative.

Solid tumors, notably those characterized by hyperfibrosis, present a formidable challenge, particularly concerning CAFs, a diverse cellular entity. The primary obstacle stems from the intricate separation task, needing more definitive markers. It should be more focused on tracking from the stage of tumorigenesis in future studies, considering CAFs as a dynamic cell with continuous developmental mutations and comparing protein expression, secretion function, and metabolic substances of CAFs in each stage of the tumors to extract substances with apparent differences. Then, we will explore the regulation of this substance by upstream and downstream molecules, which may lead to a deeper understanding of the role played by CAFs in tumor progression.

In clinical practice, given that inflammatory factors orchestrate most of the pro-tumorigenic effects attributed to CAFs, it is paramount to consider the management of inflammation as a critical aspect of treatment. In the surgical treatment strategy, does the non-tumor part of the cancerous organ contain CAFs? Whether the "wound" caused by the removal of a local tumor, which triggers local inflammation and an increase in inflammatory factors in the blood, will stimulate the transformation of normal fibroblasts in the cancerous organ into CAFs or activate the activity of remaining CAFs leading to recurrence, is a question worth deep consideration. Whether to intervene or add immunosuppressive agents for the increase of immune cells after cancer surgery remains to be determined by clinical practice.

In conclusion, to gain a comprehensive grasp of the remarkable plasticity and dynamic instability exhibited by CAFs, it will be helpful to delve deeper into the diverse array of anti-cancer CAFs and their underlying mechanisms of action. This includes investigating whether various subtypes of CAF exhibit similar characteristics across different stages of cancer progression and microenvironment and whether the crosstalk mechanism remains consistent among all components. Meanwhile, improving the communication mechanism within the CAFs relationship network and inducing CAFs to transition into a resting state may be more reasonable than indiscriminately eliminating CAFs. Finally, although the mechanisms of action within tumors are complex with numerous therapeutic entry points, decentralized exploration should also consider commonalities between these mechanisms. Indeed, the survival of patients with solid cancers is expected to improve through combinations of therapies.

## Data Availability

Not applicable
